# Temporal variations in reference evapotranspiration in the Tarim River basin, Central Asia

**DOI:** 10.1371/journal.pone.0252840

**Published:** 2021-06-16

**Authors:** Hao Wu, Min Xu, Zhuoyue Peng, Xiaoping Chen

**Affiliations:** 1 College of Hydraulic Science and Engineering, Yangzhou University, Yangzhou, China; 2 State Key Laboratory of Cryospheric Science, Northwest Institute of Eco-Environment and Resources, Chinese Academy of Sciences, Lanzhou, China; Soil and Water Resources Institute ELGO-DIMITRA, GREECE

## Abstract

Reference evapotranspiration (ET_0_) is important for agricultural production and the hydrological cycle. Knowledge of ET_0_ can aid the appropriate allocation of irrigation water in arid regions. This study analyzed the trends in ET_0_ over different timescales in the Tarim River basin (TRB), Central Asia. ET_0_ was calculated by the Penman-Monteith method using data from 1960–2017 from 30 meteorological stations located in the TRB. The Mann-Kendall (MK) test with trend-free prewhitening and Sen’s slope estimator were applied to detect trends in ET_0_ variation. The results showed that the mean ET_0_ decreased at a rate of 0.49 mm·10 a^-1^ on an annual timescale. The mean ET_0_ exhibited a decreasing trend in summer and increasing trends in other seasons. The effects of climatic factors on ET_0_ were assessed by sensitivity analysis and contribution rate analysis. Maximum temperature (T_max_), relative humidity (RH) and wind speed (WS) showed important effects on ET_0_. However, WS, which decreased, was the key element that induced changes in ET_0_ in the TRB. This work provides an important baseline for the management of agricultural water resources and scientific planning in agriculture.

## Introduction

Evapotranspiration (ET) is an important part of the water cycle; it controls energy exchange in ecological systems and has been a focus of studies on water resources, agriculture and ecosystems [1]. ET can be observed by various methods, including eddy covariance systems, large aperture scintillometer analysis, water balance methods, micrometeorological methods and lysimeter analysis [2–4]. However, in actually irrigation and drainage planning, ET is usually calculated by the crop coefficient method, and reference evapotranspiration (ET_0_) is a key indicator in this approach [5]. ET_0_ is the evapotranspiration from a reference surface that is similar to extensive grasslands of uniform height and vigorous growth that are supplied with well water. The Penman-Monteith formula is a fairly reliable method for estimating ET_0_ and is widely used [6–8].

ET_0_ represents the potential evaporation of the atmosphere [9] and is affected by climate factors, such as solar radiation, wind speed (WS), precipitation and air temperature [10]. A previous study has shown that the mean temperature near the surface of the earth increased by 0.74°C in the twentieth century [11]. As air temperature increases, evapotranspiration from land-based ecosystems increases. However, observations have shown that in many areas, ET_0_ has decreased. This combination of global temperature increases and ET_0_ decreases is called the pan evaporation paradox [12]. This paradox and the key factors responsible have been widely discussed in many studies. For example, in the Platte River Basin, central Nebraska, USA, ET_0_ showed a decreasing trend under changing precipitation [13]. In humid and desert regions in India, ET_0_ showed a decreasing trend under changing net radiation and WS [14, 15]. In the Yangtze River, Yellow River, Meigong River and Gan-Xin Region of China, ET_0_ exhibited a decreasing trend, and the main influencing factors were radiation, sunshine hours (SH) and WS [8, 16–21].

The Tarim River basin (TRB) is situated inland and has a dry climate, low rainfall, strong evaporation, water shortages, and an extremely fragile ecological environment. The upper reaches of the basin are mountainous areas and oases, and the lower reaches are deserts [22]. The water cycle in the basin is characterized by water production in mountainous areas [23] and water consumption in oasis areas. The basin is an important irrigated agricultural area. With the development of agriculture in the oasis regions, the farmland area and irrigation water consumption have increased. An investigation showed that irrigation water consumption accounts for more than 98% of the total water consumption [24]. Increasing irrigation water use and intensive evaporation have caused serious secondary salinization of the soil. Approximately 48% of farmland in the TRB exhibits some degree of soil salinization. In addition, the increase in irrigated agriculture has led to reductions in the discharge of natural rivers and declines in the natural vegetation in desert areas [25]. The TRB is the core area of the Silk Road Economic Belt and plays an important strategic role in the overall opening-up of China. Continued economic development must rely on water resources, and any change in the water cycle will significantly impact agricultural and hydrological processes. Evapotranspiration has an important influence on the amount of available water resources in the TRB. Thus, changes in the spatiotemporal distribution of ET_0_ will alter the amount of available water resources in this region.

Previous studies of ET_0_ have focused on large agricultural regions, with ET_0_ calculated as the average over each of these areas [20, 26]. ET_0_ trends at different weather stations in the TRB have not been detected. Investigating the influence of climate change on ET_0_ can help guide regional water resource management and the adjustment of cropping structures. The main purposes of this paper are to (1) explore changes in the spatial and temporal distributions of ET_0_ in the TRB and (2) analyze the key meteorological elements affecting ET_0_.

## Materials and methods

### Study area and data

**Fig 1:**
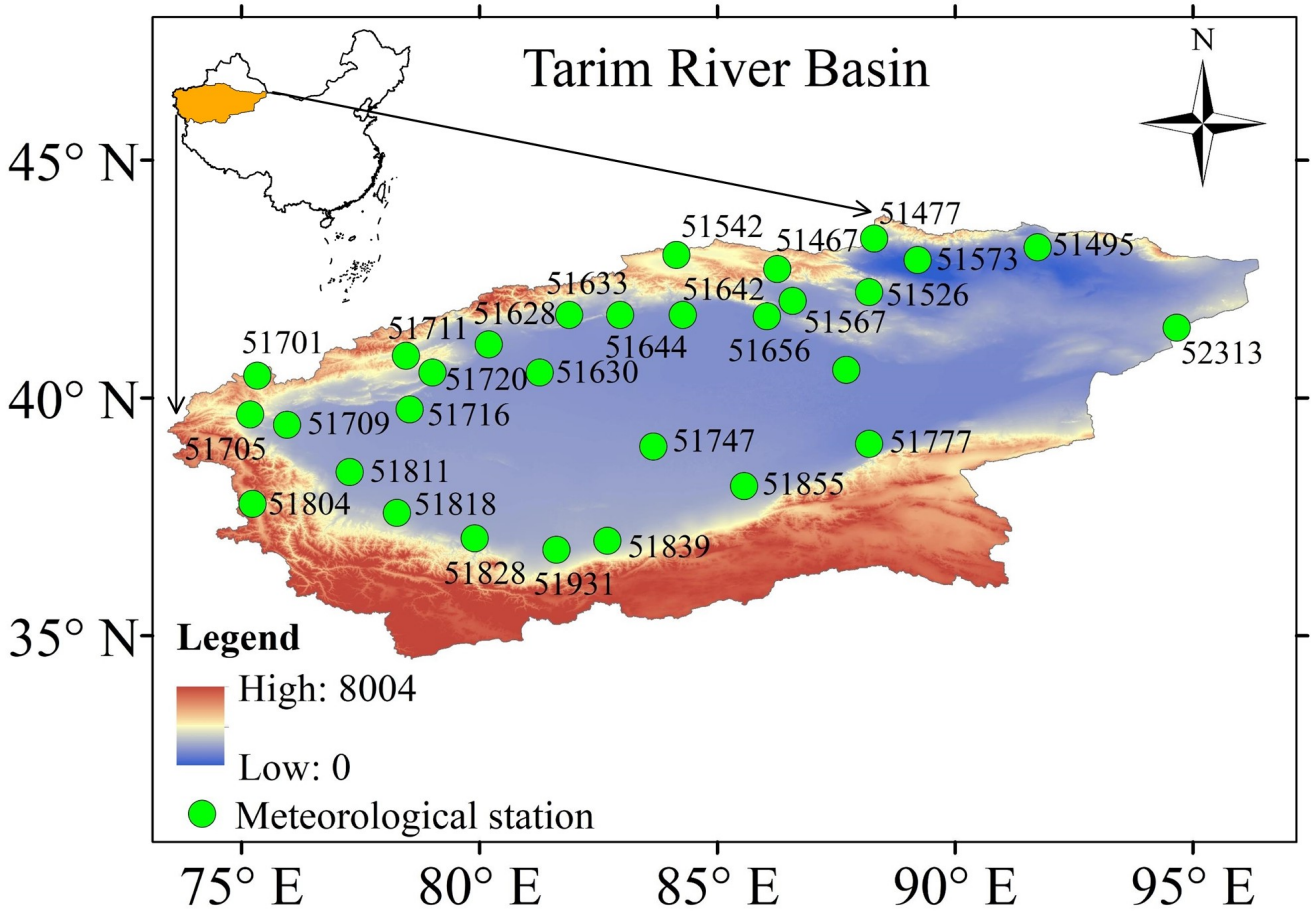
Locations of the meteorological stations involved in this study.

The TRB is located in Central Asia (34°-45° N; 73°-97° E) and covers a total area of approximately 1.02×106 km^2^ (Fig 1). It is surrounded by Tianshan, eastern Pamir, Kunlun, and the Karakorum Mountains, with elevations ranging from -156 m to 8238 m [27]. The precipitation is greater than 300 mm in the mountainous regions and below 50 mm in the lower basin. More than 54% of the precipitation occurs in June, July and August. The temperature ranges from -35°C in winter to 40°C in summer [28]. The water resources of the basin are mainly generated by rainfall and glacier/snow meltwater in the mountainous regions [29]. The oasis is the main social unit in the TRB, and agriculture is an important component of oasis sustainable development. The water consumption of agricultural production amounts to 98% of the total water consumption in the TRB.

In this study, climate data for the period of 1960~2017 from 30 meteorological stations distributed all over the TRB were obtained from the China Meteorological Data Service Center (http://data.cma.cn/) (Table 1). The data included maximum and minimum temperature (T_max_, T_min_), relative humidity (RH), wind speed (WS), and sunshine hour (SH) on a daily timescale. The daily ET_0_ values were estimated from these five meteorological elements, and seasonal and annual ET_0_ were estimated from daily ET_0_.

**Table 1:** Meteorological stations involved in this study.

ID	Station	Longitude (°E)	Latitude (°N)	Elevation (m)	Average temperature (°C)
51467	Baluntai	86.30	42.73	1739	7.39
51477	Dabancheng	88.32	43.35	1103.5	7.14
51495	Qijiaojing	91.73	43.22	721.4	10.71
51526	Kumishi	88.22	42.23	922.4	9.55
51542	Bayinbuluke	84.15	43.03	2458	-3.63
51567	Yanqi	86.57	42.08	1055.3	9.08
51573	Yulufan	89.20	42.93	34.5	15.27
51628	Akesu	80.23	41.17	1103.8	11.12
51633	Baicheng	81.90	41.78	1229.2	8.69
51642	Luntai	84.25	41.78	976.1	11.73
51644	Kuche	82.97	41.72	1081.9	11.63
51656	Kuerle	86.13	41.75	931.5	12.11
51701	Tuergate	75.40	40.52	3504.4	-2.80
51705	Wuqia	75.25	39.72	2175.7	7.82
51709	Kashi	75.98	39.47	1289.4	12.23
51711	Aheqi	78.45	40.93	1984.9	7.35
51716	Bachu	78.57	39.80	1116.5	12.47
51720	Keping	79.05	40.50	1161.8	11.91
51730	Alaer	81.27	40.55	1012.2	11.35
51747	Tazhong	83.67	39.00	1099.3	11.95
51765	Tieganlike	87.70	40.63	846	11.66
51777	Ruoqiang	88.17	39.03	887.7	12.29
51804	Tashikuergan	75.23	37.77	3090.1	3.90
51811	Shache	77.27	38.43	1231.2	12.34
51818	Pishan	78.28	37.62	1375.4	12.72
51828	Hetian	79.93	37.13	1375	13.37
51839	Minfeng	82.72	37.07	1409.5	12.16
51855	Qiemo	85.55	38.15	1247.2	11.09
51931	Yutian	81.65	36.85	1422	12.33
52313	Hongliuhe	94.67	41.53	1573.8	7.03

### Methods

#### Penman-Monteith method

ET_0_ is the evapotranspiration from a reference surface with a height of 0.12 m, an albedo of 0.23 and a surface resistance of 70 s/m [9]. The Penman-Monteith equation has been recommended as the standard method for determining ET_0_ by the FAO [30]. The equation is shown below:

$$ET_{0}=\frac{0.408(R_{n}-G)+\gamma \frac{900}{T+273}u_{2}(e_{a}-e_{d})}{\Delta +\gamma (1+0.34u_{2})}$$ (1)

where *ET*_0_ is the reference evapotranspiration (mm·m^-2^·day^-1^), *R*_*n*_ is the net radiation (MJ·m^-2^·day^-1^), G is the soil heat flux (MJ·m^-2^·day^-1^), *γ* is the psychrometric constant (k·Pa·°C^-1^), *T* is the air temperature (°C), *u*_2_ is the wind speed at a height of 2 m (m·s^-1^), *e*_*a*_ is the saturated vapor pressure (kPa), *e*_*d*_ is the actual vapor pressure (kPa), and Δ is the slope of the saturation vapor pressure curve (kPa·°C^-1^).

#### Mann-Kendall test with trend-free prewhitening

In the Mann-Kendall (MK) test with trend-free prewhitening (TFPW-MK), the influence of the trend on the autocorrelation coefficient was removed, allowing the MK test to be performed on the data sequence with greater accuracy; this approach has been widely used in studies of hydrological trend detection [31–33]. The equations for the MK test method are given below:

$$S=\sum_{k=1}^{n-1}\sum_{j=k+1}^{n}Sgn(S_{j}-S_{k})$$ (2)

$$Sgn(S_{j}-S_{k})=\{\begin{array}{l} 1\;(X_{j}-X_{k})>0\\ 0\;(X_{j}-X_{k})=0\\ -1\;(X_{j}-X_{k})<0 \end{array}$$ (3)

$$Var(S)=\frac{n(n-1)(2n+5)-\sum_{i=1}^{m}t_{i}(t_{i}-1)(2t_{i}+5)}{18}$$ (4)

$$Z=\{\begin{array}{l} \frac{S-1}{\sqrt{Var(S)}}\;S>0\\ 0\;S=0\\ \frac{S+1}{\sqrt{Var(S)}}\;S<0 \end{array}$$ (5)

where *X*_*i*_ is the value of year *i*; *n* is the length of the data, and *m* is the number of groups with tied ranks, each with *t*_*i*_ tied observations. If |*Z*|≥*Z*_1-*α*/2_, the null hypothesis is rejected, and the alternative hypothesis is accepted at the significance level of *α*; otherwise, the null hypothesis of no trend is accepted at the significance level of *α*.

Trend-free prewhitening includes the following steps:

$$r_{1}=\frac{\sum_{t=1}^{n-1}\left(x_{t}-\bar{x_{t}})(x_{t+1}-\bar{x_{t+1}}\right)}{\sqrt{\sum_{t=1}^{n-1}{\left(x_{t}-\bar{x_{t}}\right)}^{2}\sum_{t=1}^{n-1}{\left(x_{t+1}-\bar{x_{t+1}}\right)}^{2}}}$$ (6)

$$Y_{t}-x_{t}-\beta t$$ (7)

$$Y_{t}^{'}=Y_{t}-r_{1}Y_{t-1}+\beta t$$ (8)

where *x*_*t*_ is the value of year *t* of the time series, *n* is the length of the data, and *___x*_*t*_ is the average value. The original MK test is applied to *Y*_*t*_ to assess the significance of the trend.

#### Sen’s slope estimator

Sen’s slope estimation is a method for estimating the magnitude of time series data [34]. In this study, the magnitudes of the trends in ET_0_ were investigated using Sen’s slope estimator.


$$\beta =Median(\frac{x_{j}-x_{i}}{j-i})\;j>i$$ (9)

If *β*> 0, the time series of *ET*_0_ and other climatic factors are increasing; otherwise, the time series are decreasing.

#### Sensitivity analysis

The sensitivity coefficient is the rate of variation in ET_0_ with meteorological elements [35, 36]. The equation is shown below:

$$Sv_{i}=\underset{v_{i}\to 0}{\lim}(\frac{\Delta ET_{0}/ET_{0}}{\Delta v_{i}/v_{i}})=\frac{\partial ET_{0}}{\partial v_{i}}\cdot \frac{\left|v_{i}\right|}{ET_{0}}$$ (10)

where *Sv*_*i*_ is the sensitivity coefficient of *v*_*i*_, Δ*ET*_0_ is the variation in *ET*_0_, *v*_*i*_ is the meteorological factor, and Δ*v*_*i*_ is the variation in *v*_*i*_. To evaluate the influence of a climate factor on ET_0_, the sensitivity coefficient was divided into four levels [37], as shown in Table 2.

**Table 2:** Classification of the sensitivity coefficient.

Sensitivity coefficient	Sensitivity level
0.00≤|*Sv*_*i*_|<0.05	Negligible
0.05≤|*Sv*_*i*_|<0.20	Moderate
0.20≤|*Sv*_*i*_|<1.00	High
1.00≤|*Sv*_*i*_|	Very high

#### Contribution rate analysis

The contribution rate of the meteorological elements is calculated by multiplying the sensitivity coefficient by its relative change rate [36]. The equations of the contribution rate of meteorological elements are as follows:

$$Conv_{i}=Sv_{i}\times RCv_{i}$$ (11)

$$RCv_{i}=\frac{n\times Trend_{v_{i}}}{\left|av_{i}\right|}\times 100$$ (12)

where *Conv*_*i*_ is the contribution rate of *v*_*i*_, *RCv*_*i*_ is the relative change rate in *v*_*i*_, *n* is the number of years, *av*_*i*_ is the mean value of *v*_*i*_, and *Trend*_*vi*_ is the annual trend in *v*_*i*_.

## Results

### Temporal variations in meteorological elements

**Fig 2:**
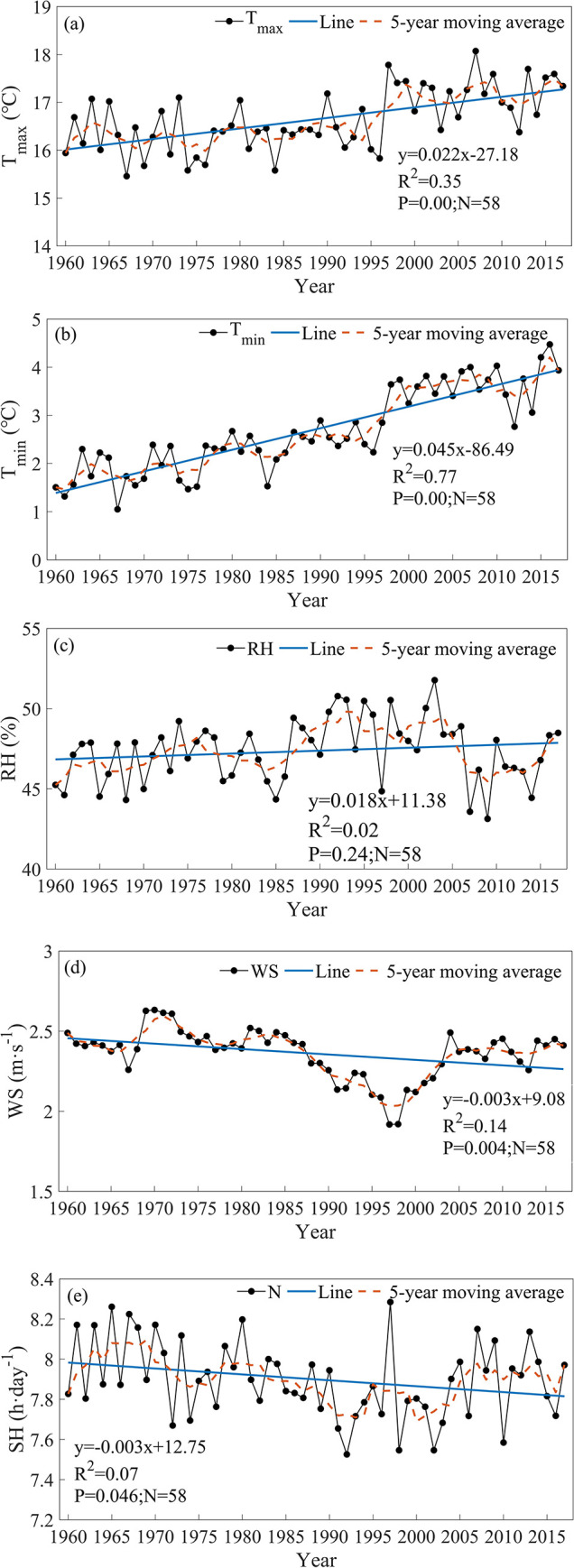
Change trends in the mean meteorological elements in the Tarim River basin during 1960–2017. (a) T_max_. (b) T_min_. (c) RH. (d) WS. (e) SH.

The TFPW-MK test and Sen’s slope estimator showed significant increasing trends in T_max_ and T_min_ and an increasing trend in RH. The rates of increase in T_max_, T_min_ and RH in the TRB were 0.21°C·10 a^−1^, 0.45°C·10 a^−1^ and 0.22%·10 a^−1^, respectively. WS and SH showed significant downward trends in the TRB, with decrease rates of -0.03 m·s^−1^·10 a^−1^ and -0.03 h·10 a^−1^, respectively (Fig 2 and Table 3).

**Table 3:** Results of the trend analyses of annual meteorological elements.

Test	T_max_	T_min_	RH	WS	SH
TFPW-MK	4.21*	7.76*	1.19	-3.90*	-2.14*
Sen’s slope estimator	0.21 (°C·10 a^-1^)	0.45 (°C·10 a^-1^)	0.22 (%·10 a^-1^)	-0.03 (m·s^-1^·10 a^-1^)	-0.03 (h·10 a^-1^)

*α < 0.05

### Temporal and spatial variability of ET_0_

#### Temporal variability of annual and seasonal ET_0_

**Fig 3:**
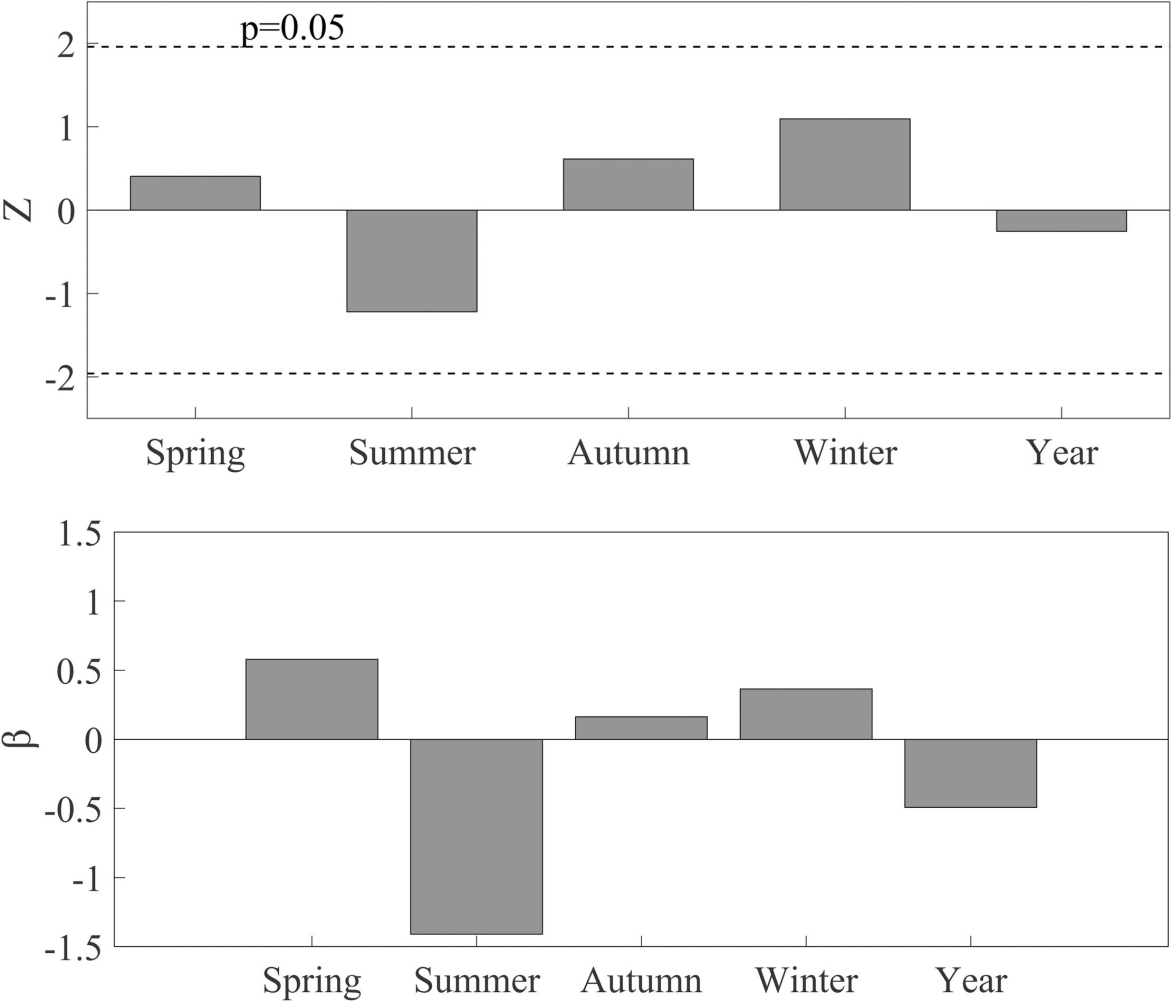
Results of the TFPW-MK test and Sen’s slope estimator for the average ET_0_ in the TRB from 1960 to 2017.

The TFPW-MK test showed seasonal differences in the mean ET_0_ trends. ET_0_ had increasing trends in spring, autumn, and winter and a decreasing trend in summer. Sen’s slope estimator showed that the rate of increase in ET_0_ in spring, autumn and winter ranged from 0.16 to 0.58 mm ·10 a^−1^. In summer, the rate of decrease in ET_0_ was 1.41 mm·10 a^-1^. The annual results of the TFPW-MK test showed that ET_0_ had a decreasing trend in the TRB, with a rate of decrease of 0.49 mm·10 a^-1^ (Fig 3).

The trend in ET_0_ varied among different sites with different microclimatic characteristics. In spring, summer, and autumn, the number of sites with decreasing trends was larger than that with increasing trends. Among the sites, 53.3%~66.7% showed decreasing trends, of which 75.0~76.5% showed significantly negative trends (Table 4). Increasing trends were observed for 33.3~47.7% of the sites, of which 50.0~60.0% showed significantly positive trends (Table 4). In winter, the number of sites with increasing trends was larger than that with decreasing trends. Among the sites, 56.7% exhibited increasing trends, of which 35.3% exhibited significant increasing trends. Furthermore, 43.3% of the sites exhibited decreasing trends, of which 46.2% exhibited significant decreasing trends (Table 4).

**Table 4:** Percent of stations with positive (significant at the 0.05 level) and negative (significant at the 0.05 level) trends in *ET*_0_ from 1960 to 2017 in the TRB.

	Spring	Summer	Autumn	Winter	Year
Trend	U	D	U	U	D
Positive	46.7% (50%)	33.3% (60%)	43.3% (54.8%)	56.7% (35.3%)	40% (66.7%)
Negative	53.3% (75%)	66.7% (75%)	56.7% (76.5%)	43.3% (46.2%)	60% (29.2%)

Note: U indicates that the regional trends are increasing; D indicates that the regional trends are decreasing.

On an annual scale, the number of sites with decreasing trends was larger than that with increasing trends; 40% of the sites exhibited increasing trends, with significant increasing trends observed for 66.7% of those sites. In addition, 60% of the sites showed decreasing annual trends, of which 29.2% exhibited significant decreasing trends (Table 4).

#### Spatial variations in ET_0_

**Fig 4:**
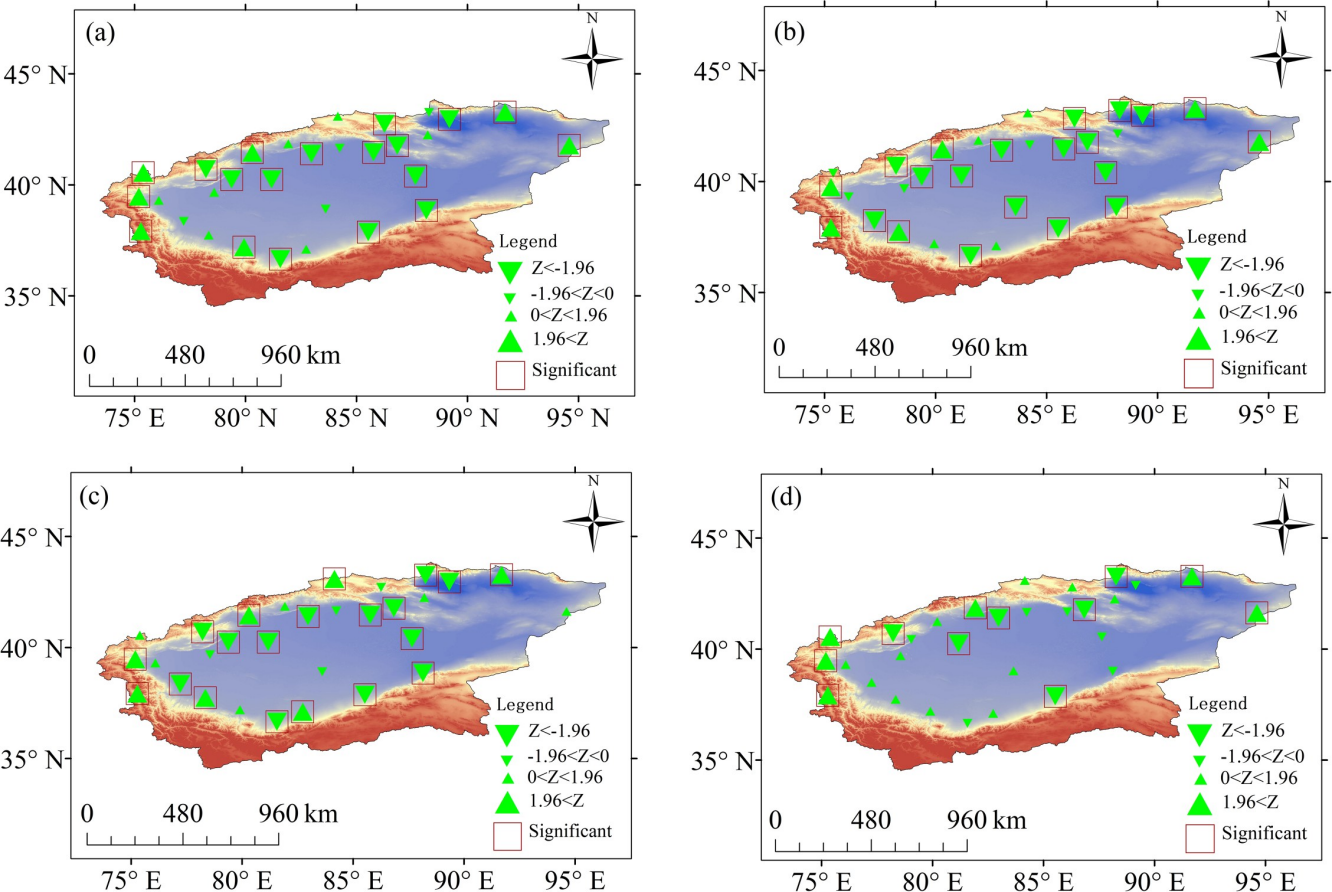
Trends in ET_0_ on seasonal timescales at different sites. (a) Spring. (b) Summer. (c) Autumn. (d) Winter.

In spring and summer, the sites with significant increasing trends were located in the eastern and western parts of the basin, those with significant decreases were localized in the southern and northern parts, and those with nonsignificant changes were spread throughout the basin (Fig 4A and 4B). In autumn, the sites with significant increasing trends or nonsignificant changes were distributed throughout the basin, whereas those with significant decreases were located in the southern and northern regions (Fig 4C). In winter, the sites with significant increasing trends were located in the eastern, northern and western regions of the basin, whereas those with significant decreases were located in the southern and northern parts. The sites with nonsignificant changes were distributed across the basin (Fig 4D).

**Fig 5:**
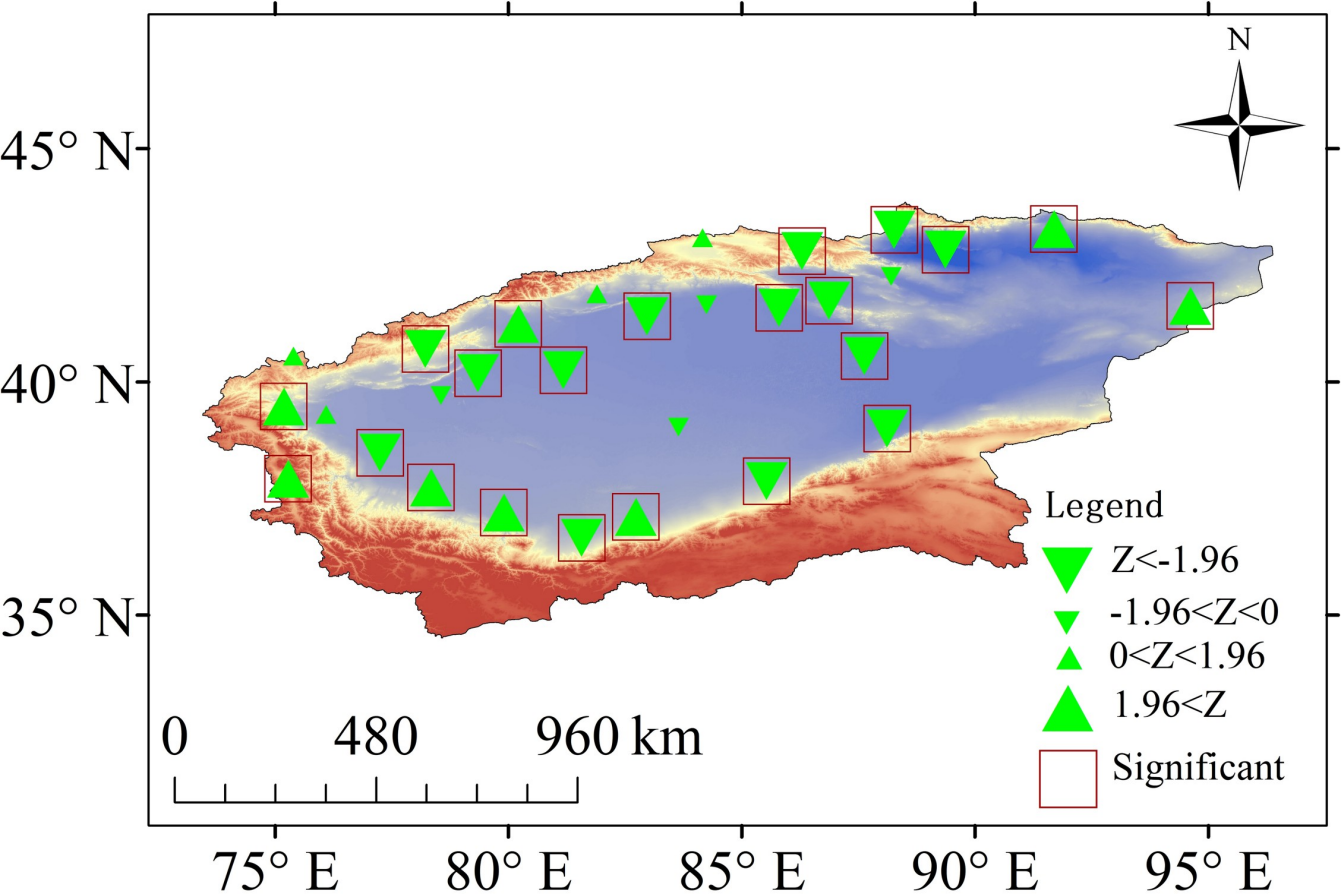
Annual trends in ET_0_ at different sites.

The sites with significant increasing trends at the annual scale were located in the eastern and western regions of the TRB. Those with significant decreases were located in the southern and northern regions of the basin (Fig 5).

### Climate factors that affect ET_0_ variability

#### Sensitivity coefficients of meteorological elements to ET_0_

Meteorological elements represent important input data for calculating ET_0_, and changes in these elements have important impacts on ET_0_. To analyze the effects of climate elements on ET_0_, sensitivity analysis was used.

The results of the sensitivity analysis showed that the average ET_0_ over the whole basin was most sensitive to T_max_, followed by RH, WS, SH and T_min_; the sensitivity coefficients were 0.354, -0.338, 0.264, 0.158 and 0.102, respectively (Table 5). The sensitivity coefficients of T_max_, RH and WS were high, whereas those of SH and T_min_ were moderate.

**Table 5:** Contribution rates of the mean meteorological elements to ET_0_.

	T_max_	T_min_	RH	WS	SH
Sensitivity coefficient	0.354	0.102	-0.338	0.264	0.158
Trend	0.021	0.038	0.033	-0.004	-0.002
Relative variation ratio	9.103	98.769	3.302	-18.534	-1.954
Contribution rate	2.828	2.314	-1.400	-4.880	-0.261

**Fig 6:**
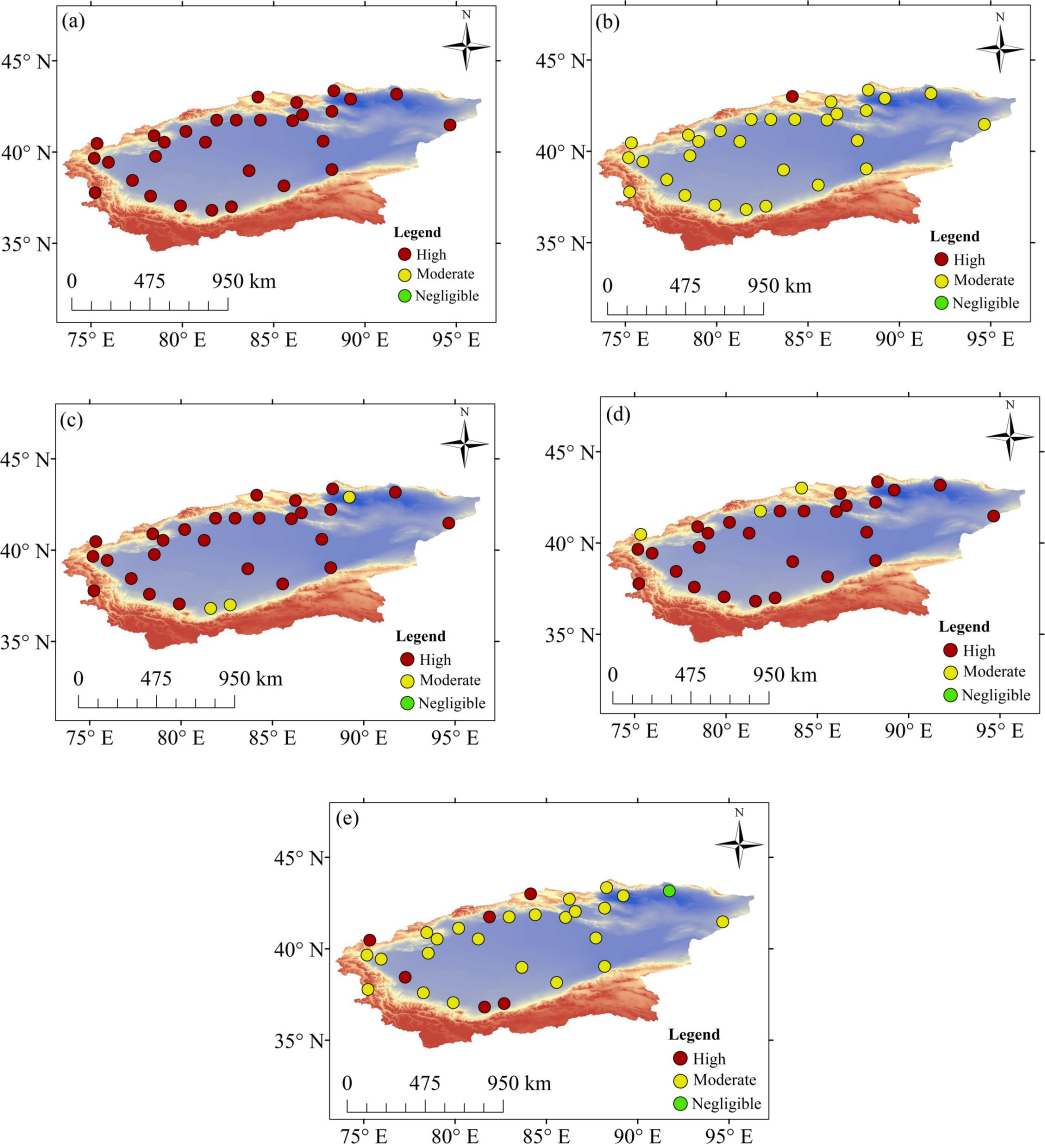
Spatial distributions of the sensitivity coefficients of meteorological elements in the TRB. a) T_max_. b) T_min_. c) RH. d) WS. e) SH.

The sensitivity coefficients varied among sites with different microclimate characteristics. The sensitivity coefficients of T_max_ were high at all sites (Fig 6A). The sensitivity coefficients of T_min_ were high at one site in the northern part of the basin and moderate at other sites (Fig 6B). For RH, the sensitivity coefficients were moderate at one site in the northeast and two sites in the south and high elsewhere (Fig 6C). The sensitivity coefficients of WS were moderate at three sites in the northern region and high at the other sites (Fig 6D). For SH, the sensitivity coefficients were high at some sites in the northern, southern, and western regions and moderate elsewhere (Fig 6E). These results showed that T_max_, RH and WS had important effects on ET_0_.

#### Contribution rates of meteorological elements to ET_0_

To explore the meteorological elements with key contributions to changes in ET_0_, contribution rate analysis was performed. The results suggested that over the whole basin, the increases in T_max_ and T_min_ positively contributed to the increase in ET_0_. In addition, increases in RH and decreases in WS and SH negatively contributed to the increase in ET_0_ (Table 5).

**Fig 7:**
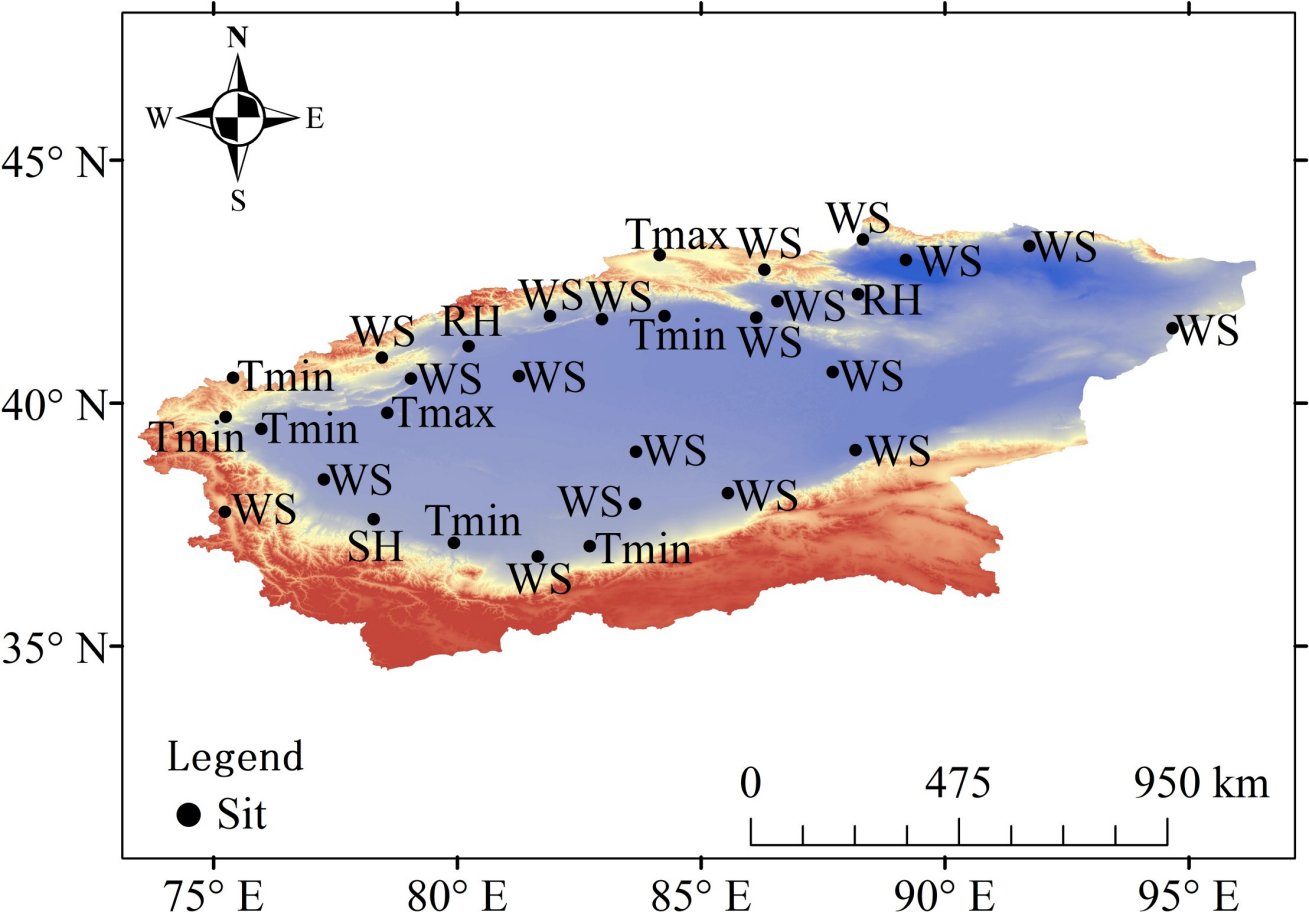
Spatial distribution of the main key meteorological elements contributing to ET_0_ in the TRB.

The contributions of meteorological elements to ET_0_ differed among the different sites. At eight sites, in the northwest, southwest and northern regions, T_min_ or T_max_ was the main factor that contributed to changes in ET_0_. At two sites, one in the northwest and one in the northeast, RH was the main factor that contributed to changes in ET_0_. At other sites near or in the Taklimakan Desert, WS was the main factor that contributed to changes in ET_0_ (Fig 7). Overall, WS, which decreased over time, was the main factor that contributed to changes in ET_0_ (Table 5).

## Discussion

### Change in ET_0_

The “evaporation paradox” has been observed in many areas and widely discussed [13–15, 26, 38, 39]. Similarly, in this study, this paradox was observed in many sites in the TRB. The annual ET_0_ exhibited a decreasing trend at many sites and over the entire area. The changes in annual ET_0_ exhibited seasonal variation. Over the entire area, ET_0_ increased in spring, autumn and winter but decreased in summer. Annual ET_0_ decreased overall because the magnitude of the decrease was larger than that of the increase.

ET_0_ is needed for irrigation planning because it is a basic input for estimating crop water requirements [40]. Under a certain irrigation guarantee rate, increases in ET_0_ will generally increase irrigation quotas and irrigation project budgets. In spring and summer, the sites with significant increasing trends were located in the eastern and western parts of the basin. Increases in ET_0_ may contribute to increased use of irrigation water by increasing water loss from farmland, thus posing a threat to crop production. The sites with significant decreases were located in the southern part of the basin. Decreases in ET_0_ may contribute to decreases in irrigation water use by reducing water loss from farmland, thus benefitting crop production. In autumn and winter, crops are harvested, and no new crops are planted in those seasons. Thus, changes in ET_0_ will have little effect on agricultural production and irrigation water.

### Climate factors that affect ET_0_

ET_0_ represents the evaporation demand of the atmosphere and is mainly affected by climatic factors [41]. Wang, Ye [20] showed that specific humidity plays the predominant role in the sensitivity of ET_0_ in Northwest China. In this study, the sensitivity analysis indicated that for the entire TRB, the largest absolute value of the sensitivity coefficient was between T_max_ and ET_0_ (Table 5). T_max_ may be the most sensitive climatic variable influencing ET_0_. The absolute values of the sensitivity coefficient of RH and ET_0_ and of WS and ET_0_ were the second and third largest, respectively. RH and WS may be the second and third most influential factors affecting ET_0_. This result may be due to the small study area, where the main landform is deserts.

The contribution rate analysis suggested that in the TRB, increasing T_max_ and T_min_ positively contributed to ET_0_, whereas increasing RH and decreasing WS and SH negatively contributed to ET_0_ (Table 5). The absolute value of the contribution of WS was greater than those of the other meteorological elements, indicating that WS was the main factor that induced changes in ET_0._ Wind plays an important role in the evapotranspiration of ecosystems. The process of vapor removal depends to a large extent on wind [9]. The study area is situated inland and experiences a dry climate and strong evaporation. Under these arid conditions, decreases in WS may result in large variations in evapotranspiration. Thomas [42] and Wang, Xie [26] emphasized that WS plays the predominant role in influencing ET_0_ in water-limited areas of Western China. The factors driving deceases in WS are extremely complex. Zhang, Ren [43] noted that changes in large-scale atmospheric circulation patterns were possible factors driving WS reduction in China. Jiang, Luo [44] found that in China, changes in WS may be driven by the East Asian winter and summer monsoons under the background of global warming. Zheng, Li [45] posited that the influence of human activities cannot be ignored and that these activities are likely the main factors driving decreases in WS in northwestern China. Liu, Dong [46] found that changes in WS in the Taklimakan Desert, Northwest China, were mainly caused by a decreased frequency of strong winds, precipitation, and urban development. However, the relationships among atmospheric circulation patterns, climate warming and human activities are extremely complex and influence each other. Changes in WS may be driven by a combination of changes in atmospheric circulation patterns, climate warming and human activities.

## Conclusions

The ET_0_ at 30 meteorological stations located in the TRB was calculated by the Penman-Monteith formula. The TFPW-MK test and Sen’s slope estimator were used to study changes in ET_0_. Sensitivity analysis and contribution rate analysis were used to identify the effects of meteorological elements and the key meteorological elements affecting ET_0_. The following main conclusions were drawn from this study.

The TFPW-MK test and Sen’s slope estimator showed seasonal differences in ET_0_ trends. The mean ET_0_ exhibited an increasing trend in spring, fall and winter and a decreasing trend in summer. On an annual timescale, the mean ET_0_ decreased, at a rate of 0.49 mm·10 a^-1^. Sensitivity analysis showed that T_max_, RH and WS had important effects on ET_0_. Contribution rate analysis showed that WS, which decreased, was the main factor that contributed to changes in ET_0_.

## Supporting information

S1 Data The data used in the article. (XLSX)

S2 Data (XLSX)

## References

[1] Yin Y, Wu S, Chen G, Dai E. Attribution analyses of potential evapotranspiration changes in China since the 1960s. Theoretical and Applied Climatology. 2009;101(1–2):19–28. doi: 10.1007/s00704-009-0197-7

[2] Widmoser P, Wohlfahrt G. Attributing the energy imbalance by concurrent lysimeter and eddy covariance evapotranspiration measurements. Agricultural and Forest Meteorology. 2018;263:287–91. doi: 10.1016/j.agrformet.2018.09.003

[3] Nelli NR, Temimi M, Fonseca RM, Weston MJ, Thota MS, Valappil VK, et al. Micrometeorological measurements in an arid environment: Diurnal characteristics and surface energy balance closure. Atmospheric Research. 2020;234. doi: 10.1016/j.atmosres.2019.104745

[4] Yee MS, Pauwels VRN, Daly E, Beringer J, Rüdiger C, McCabe MF, et al. A comparison of optical and microwave scintillometers with eddy covariance derived surface heat fluxes. Agricultural and Forest Meteorology. 2015;213:226–39. doi: 10.1016/j.agrformet.2015.07.004

[5] Wang Y, Cai H, Yu L, Peng X, Xu J, Wang X. Evapotranspiration partitioning and crop coefficient of maize in dry semi-humid climate regime. Agricultural Water Management. 2020;236. doi: 10.1016/j.agwat.2020.106164

[6] Goyal RK. Sensitivity of evapotranspiration to global warming: a case study of arid zone of Rajasthan (India). Agr Water Manage. 2004;69(1):1–11. doi: 10.1016/j.agwat.2004.03.014 WOS:000223753300001.

[7] Dinpashoh Y, Jhajharia D, Fakheri-Fard A, Singh VP, Kahya E. Trends in reference crop evapotranspiration over Iran. J Hydrol. 2011;399(3–4):422–33. doi: 10.1016/j.jhydrol.2011.01.021 WOS:000288828500025.

[8] Gao ZD, He JS, Dong KB, Li X. Trends in reference evapotranspiration and their causative factors in the West Liao River basin, China. Agr Forest Meteorol. 2017;232:106–17. doi: 10.1016/j.agrformet.2016.08.006 WOS:000389089800009.

[9] Allen RG, Pereira LS, Raes D, Smith M. Crop evapotranspirationguide-lines for computing crop water requirements. Rome Italy: FAO irrigation and drainage paper 56; 1998.

[10] Donohue RJ, McVicar TR, Roderick ML. Assessing the ability of potential evaporation formulations to capture the dynamics in evaporative demand within a changing climate. J Hydrol. 2010;386(1–4):186–97. doi: 10.1016/j.jhydrol.2010.03.020 WOS:000278577000015.

[11] Feng S, Hu Q, Huang W, Ho C-H, Li R, Tang Z. Projected climate regime shift under future global warming from multi-model, multi-scenario CMIP5 simulations. Global and Planetary Change. 2014;112:41–52. doi: 10.1016/j.gloplacha.2013.11.002

[12] Roderick M, Farquhar G. The Cause of Decreased Pan Evaporation over the Past 50 Years. Science. 2002;298(5597):1410–1. doi: 10.1126/science.1075390 12434057

[13] Irmak S, Kabenge I, Skaggs KE, Mutiibwa D. Trend and magnitude of changes in climate variables and reference evapotranspiration over 116-yr period in the Platte River Basin, central Nebraska–USA. Journal of Hydrology. 2012;420–421:228–44. doi: 10.1016/j.jhydrol.2011.12.006

[14] Jhajharia D, Dinpashoh Y, Kahya E, Singh VP, Fakheri-Fard A. Trends in reference evapotranspiration in the humid region of northeast India. Hydrol Process. 2012;26(3):421–35. doi: 10.1002/hyp.8140 WOS:000299373000010.

[15] Jhajharia D, Kumar R, Dabral PP, Singh VP, Choudhary RR, Dinpashoh Y. Reference evapotranspiration under changing climate over the Thar Desert in India. Meteorological Applications. 2015;22(3):425–35. doi: 10.1002/met.1471

[16] Xu CY, Gong LB, Jiang T, Chen DL, Singh VP. Analysis of spatial distribution and temporal trend of reference evapotranspiration and pan evaporation in Changjiang (Yangtze River) catchment. J Hydrol. 2006;327(1–2):81–93. doi: 10.1016/j.jhydrol.2005.11.029 WOS:000239671700007.

[17] Tao XE, Chen H, Xu CY, Hou YK, Jie MX. Analysis and prediction of reference evapotranspiration with climate change in Xiangjiang River Basin, China. Water Sci Eng. 2015;8(4):273–81. doi: 10.1016/j.wse.2015.11.002 WOS:000375841500002.

[18] Zhang KX, Pan SM, Zhang W, Xu YH, Cao LG, Hao YP, et al. Influence of climate change on reference evapotranspiration and aridity index and their temporal-spatial variations in the Yellow River Basin, China, from 1961 to 2012. Quaternary International. 2015;380:75–82. doi: 10.1016/j.quaint.2014.12.037 WOS:000360653300009.

[19] Li B, Chen F, Guo HD. Regional complexity in trends of potential evapotranspiration and its driving factors in the Upper Mekong River Basin. Quaternary International. 2015;380:83–94. doi: 10.1016/j.quaint.2014.12.052 WOS:000360653300010.

[20] Wang ZZ, Ye AL, Wang LH, Liu KL, Cheng L. Spatial and temporal characteristics of reference evapotranspiration and its climatic driving factors over China from 1979–2015. Agr Water Manage. 2019;213:1096–108. WOS:000457952700103.

[21] Duethmann D, Blöschl G. Why has catchment evaporation increased in the past 40 years? A data-based study in Austria. Hydrology and Earth System Sciences. 2018;22(10):5143–58. doi: 10.5194/hess-22-5143-2018

[22] Chen Y, Hao X, Chen Y, Zhu C. Study on Water System Connectivity and Ecological Protection Countermeasures of Tarim River Basin in Xinjiang. Bulletin of Chinese Academy of Sciences. 2019;34(10):1156–64. doi: 10.16418/j.issn.1000-3045.2019.10.018

[23] Xu M, Kang S, Wu H, Yuan X. Detection of spatio-temporal variability of air temperature and precipitation based on long-term meteorological station observations over Tianshan Mountains, Central Asia. Atmospheric Research. 2018;203:141–63. doi: 10.1016/j.atmosres.2017.12.007

[24] Ma Z, Su S, Long A, Zhang X. Water Cycle Analysis of Social and Economic System in Tarim River Basin. dvances in Earth Science. 2018;33(8):833–41.

[25] Chen Y, Li W, Chen Y, Zhao R, Wan J. Ecological Response and Ecological Regeneration of Transfusing Stream Water along the Dried-up Watercourse in the Lower Reaches of the Tarim River, Xinjiang. Arid Zone Research. 2006;23(4):521–30. doi: 10.13866/j.azr.2006.04.002

[26] Wang Z, Xie P, Lai C, Chen X, Wu X, Zeng Z, et al. Spatiotemporal variability of reference evapotranspiration and contributing climatic factors in China during 1961–2013. Journal of Hydrology. 2017;544:97–108. doi: 10.1016/j.jhydrol.2016.11.021

[27] Xu M, Wang X, Sun T, Wu H, Li X, Kang S. Water balance change and its implications to vegetation in the Tarim River Basin, Central Asia. Quaternary International. 2019;523:25–36. doi: 10.1016/j.quaint.2019.06.011

[28] Tao H, Gemmer M, Bai Y, Su B, Mao W. Trends of streamflow in the Tarim River Basin during the past 50years: Human impact or climate change? J Hydrol. 2011;400(1–2):1–9. doi: 10.1016/j.jhydrol.2011.01.016

[29] Gao X, Ye B, Zhang S, Qiao C, Zhang X. Glacier runoff variation and its influence on river runoff during 1961–2006 in the Tarim River Basin, China. Science China Earth Sciences. 2010;53(6):880–91. doi: 10.1007/s11430-010-0073-4

[30] Menzel L, Burger G. Climate change scenarios and runoff response in the Mulde catchment (Southern Elbe, Germany). J Hydrol. 2002;267(1–2):53–64. Pii S0022-1694(02)00139-7 doi: 10.1016/S0022-1694(02)00139-7 WOS:000178228700006.

[31] Liu TG, Li LG, Lai JB, Liu C, Zhuang WH. Reference evapotranspiration change and its sensitivity to climate variables in southwest China. Theor Appl Climatol. 2016;125(3–4):499–508. WOS:000380703300008.

[32] Yue S, Pilon P, Phinney B, Cavadias G. The influence of autocorrelation on the ability to detect trend in hydrological series. Hydrol Process. 2002;16(9):1807–29. doi: 10.1002/hyp.1095

[33] Byakatonda J, Parida BP, Moalafhi DB, Kenabatho PK. Analysis of long term drought severity characteristics and trends across semiarid Botswana using two drought indices. Atmos Res. 2018;213:492–508. doi: 10.1016/j.atmosres.2018.07.002

[34] Pandey BK, Khare D. Identification of trend in long term precipitation and reference evapotranspiration over Narmada river basin (India). Global and Planetary Change. 2018;161:172–82. doi: 10.1016/j.gloplacha.2017.12.017

[35] McCuen RH. A sensitivity and error analysis of procedures used for estimating evaporation. J Am Water Resour As. 1974;10(3):486–97.

[36] Li C, Wu PT, Li XL, Zhou TW, Sun SK, Wang YB, et al. Spatial and temporal evolution of climatic factors and its impacts on potential evapotranspiration in Loess Plateau of Northern Shaanxi, China. Science of the Total Environment. 2017;589:165–72. doi: 10.1016/j.scitotenv.2017.02.122 WOS:000399848100018. 28258753

[37] Lenhart T, Eckhardt K, Fohrer N, Frede HG. <omparison of two different approaches of sensitivity analysis. Phys Chem Earth. 2002;27(9–10):645–54.

[38] Rayner DP. Wind Run Changes: The Dominant Factor Affecting Pan Evaporation Trends in Australia. Journal of Climate. 2007;20(14):3379–94. doi: 10.1175/jcli4181.1

[39] Gao Z, He J, Dong K, Li X. Trends in reference evapotranspiration and their causative factors in the West Liao River basin, China. Agricultural and Forest Meteorology. 2017;232:106–17. doi: 10.1016/j.agrformet.2016.08.006

[40] Cruz-Blanco M, Lorite IJ, Santos C. An innovative remote sensing based reference evapotranspiration method to support irrigation water management under semi-arid conditions. Agricultural Water Management. 2014;131:135–45. doi: 10.1016/j.agwat.2013.09.017

[41] Zhang Q, Xu C-Y, Chen X. Reference evapotranspiration changes in China: natural processes or human influences? Theoretical and Applied Climatology. 2010;103(3–4):479–88. doi: 10.1007/s00704-010-0315-6

[42] Spatial Thomas A. and temporal characteristics of potential evapotranspiration trends over China. Int J Climatol. 2000;20(4):381–96.

[43] Zhang A, Ren G, Guo J, Wang Y. Change trend analyzes on upper-air wind speed over china in past 30 years Plateau Meteorol. 2009;28(3):680–7.

[44] Jiang Y, Luo Y, Zhao Z, Tao S. Changes in wind speed over China during 1956–2004. Theoretical and Applied Climatology. 2009;99(3–4):421–30. doi: 10.1007/s00704-009-0152-7

[45] Zheng J, Li B, Chen Y, Chen Z, Lian L. Spatiotemporal variation of upper-air and surface wind speed and its influencing factors in northwestern China during 1980–2012. Theoretical and Applied Climatology. 2017;133(3–4):1303–14. doi: 10.1007/s00704-017-2346-8

[46] Liu Z, Dong Z, Zhang Z, Cui X, Xiao N. Spatial and temporal variation of the near-surface wind regimes in the Taklimakan Desert, Northwest China. Theoretical and Applied Climatology. 2019;138(1–2):433–47. doi: 10.1007/s00704-019-02824-w

